# Notes

**Published:** 1902-06

**Authors:** 


### NOTES.

PHYSICKING IN MILK FEVER CASES.—In almost every form of treatment for milk fever the prescriptions include a cathartic of some kind. In some, the physic is given the first consideration, but opinions vary as to the strength and number of doses it is advisable to give. For the past decade it has been generally accepted that the bowels of the patient must be kept active during the whole period of confinement for calving, and it would be appalling if the mountain of epsom salts giving dairy cows in that time could be displayed in one heap.

An opposite view of the case is credited to Mr. Obadiah Brown, in a bulletin lately issued by the Rhode Island State Board of Agriculture. His method of treating milk fever omits the purging process, and he is quoted as saying, "I have never known a cow treated with physic to recover from milk fever; with my treatment I have never lost a cow."

This will be rather surprising to many who have been reading about the value of physic at calving time. It may be interesting to know what Mr. Brown does instead, although his may be a familiar method. He says:

"My experience has been confined to my herd and to some of my neighbors' cows. My treatment is with laudanum and spirits of sweet nitre. When the cow is first taken I give an ounce of laudanum and nitre in a pint of blood-warm water sweetened with molasses.

"Shake up together in a quart bottle, hold up the cow's head, slip the neck of the bottle in the side of her mouth, between the grinders and the front teeth and let the liquid run down her throat. If this does not relieve her she will bloat slightly and appear uneasy. In three or four hours give one-half ounce more of laudanum and nitre; repeat this dose as often as she becomes uneasy, or in three or four hours. If this does not relieve the cow, increase the quantity until the medicine masters the disease.

"One of my cows had milk fever three years in succession. The ordinary dose did not relieve her. I gave two ounces of laudanum and two of nitre at one dose. It had the desired effect, and relieved her so that in a few hours she was on her feet eating hay."—*Jersey Bulletin.*

Veterinary Surgeon Desmond, of Adelaide, South Australia, reports a case of trichinosis in man. A cancerous-like growth of the neck on a patient proved on careful microscopical examination to be filled with numerous specimens of the trichina spiralis. In an article published in the Adelaide *Advertiser*, Dr. Desmond reviews the literature and the method of its development, etc.

Twenty years' practice with high class stock is the way a Long Island veterinarian announces himself to the public.

